# Deep and durable response to re-challenge with platinum-based therapy in a platinum-relapsed small cell lung cancer patient following Olvi-Vec oncolytic immunotherapy: a case report

**DOI:** 10.3389/fonc.2026.1923418

**Published:** 2026-09-09

**Authors:** Yun Chen, Zhi-Quan Qin, Zi-Ming Li, Xing-Hao Ai, Yong A. Yu, Ralph Smalling, Hannah Chen, Helena Zhu, Shun Lu

**Affiliations:** 1 Cancer Center, Department of Medical Oncology, Zhejiang Provincial People’s Hospital, Hangzhou, China; 2 Shanghai Lung Cancer Center, Shanghai Key Laboratory of Thoracic Tumor Biotherapy, Shanghai Chest Hospital, Shanghai Jiao Tong University School of Medicine, Shanghai, China; 3 Genelux Corporation, Westlake Village, CA, United States; 4 Newsoara HYK Biopharmaceutical (Shanghai) Co., Ltd., Shanghai, China

**Keywords:** case report, modified vaccinia virus, Olvi-Vec, oncolytic immunotherapy, platinum rechallenge, refractory cancer, relapsed cancer, small cell lung cancer (SCLC)

## Abstract

Oncolytic viral therapy has broad antitumor and immuno-oncology effects that may be useful in managing malignancies, particularly in relapsed or refractory diseases following conventional therapeutic regimens. We report a case involving a patient enrolled in an ongoing phase Ib clinical trial of Olvi-Vec, a modified oncolytic vaccinia virus, in patients with platinum-relapsed/refractory advanced small cell lung cancer (SCLC). In this 58-year-old woman who had progressed after frontline treatment with platinum and etoposide, a deep and durable partial objective response (84.6% reduction in target lesion size) and progression-free survival (16.7 months) were observed following a single course of Olvi-Vec followed by re-challenge with platinum-based therapy. These findings exceed expected clinical outcomes based on historical data. Olvi-Vec therapy was well tolerated. The clinical effect observed in this patient may be related to Olvi-Vec-mediated changes in the tumor microenvironment, potentially leading to platinum re-sensitization. The results of this case report indicate that the role of Olvi-Vec as an oncolytic immunotherapy for cancer treatment warrants additional study.

## 1 Introduction

Lung cancer is the most frequently diagnosed and deadliest cancer worldwide (1). About 15% of lung cancer cases are classified as small cell lung cancer (SCLC), which is characterized by a rapid proliferation rate, early metastasis, and high mortality. More than 80% of SCLC patients are diagnosed with stage III/IV cancer at first presentation (2).

For many years, the recommended regimen of first-line therapy for advanced SCLC has been platinum-based; more recently, immunotherapeutic agents, particularly the programmed death 1 (PD-1) and programmed death ligand 1 (PD-L1) inhibitors, are being added to first-line chemotherapy (3). Although most patients experience an initial response to treatment, chemoresistance develops rapidly, and median overall survival in patients with advanced SCLC is approximately 9 to 15 months (4, 5). Effective treatment of this aggressive disease is thus a significant unmet medical need.

Patients with platinum-relapsed or -refractory SCLC have a particularly poor prognosis. Due to the poor clinical outcomes with currently available systemic agents, clinical trial enrollment is a preferred option for patients with SCLC who fail first-line therapy (3). For patients treated outside of clinical trials, American Society of Clinical Oncology (ASCO) (6) guidelines recommend second-line therapy based on the duration of the chemotherapy-free interval (CTFI) following first-line platinum-based regimens, defined as the time between the last dose of chemotherapy and the first sign of disease progression. For patients with a CTFI ≥3 months, ASCO guidelines recommend platinum re-challenge (6). National Comprehensive Cancer Network (NCCN) guidelines recently removed specific CTFI-based recommendations, but list rechallenge with platinum-based regimens as a preferred option for patients with a “prolonged disease-free time” following initial platinum-based therapy (3). This recommendation is based in part on a randomized phase III trial comparing carboplatin + etoposide to topotecan in patients with disease progression after an initial response to platinum therapy (7), which found a significantly longer progression-free survival (PFS) in the platinum arm (4.7 months vs 2.7 months; p = 0.004). Other agents listed by guidelines for SCLC patients following failure of platinum-based regimens include tarlatamab-dlle, irinotecan, lurbinectedin, and topotecan (3, 6). The role of immune checkpoint inhibitors in patients who have failed first-line therapy remains unclear (8). Regardless of the second-line regimen used, treatment failure typically occurs within a few months (9).

Multiple mechanisms potentially related to chemotherapy resistance have been identified in SCLC cells, including alterations in the apoptotic cascade, changes in drug influx and efflux, increased drug metabolism, and enhanced immune evasion (10, 11). Although many new therapeutic agents and treatment modalities are being urgently explored (8), the significant percentage of patients who develop chemotherapy resistance remains an ongoing challenge in the clinical management of SCLC.

Given the current incremental improvements in treating advanced SCLC, novel approaches to therapy are required to improve outcomes in this disease, particularly in patients with relapsed or refractory SCLC. One such approach is oncolytic viral immunotherapy, in which genetically modified viruses replicate within and kill cancer cells through immunogenic cell death (ICD), resulting in robust immune activation and immune memory against a wide range of cancers (12). Oncolytic viruses also have the advantage of using different modes of cellular entry and having alternative antitumor mechanisms from conventional drugs, which may provide new treatment options for refractory/resistant cancers (12). Vaccinia virus is a particularly attractive candidate for oncolytic therapy due to its tumor selectivity, absence of integration into host DNA, strong antitumor activity, and immune activation and memory (13).

Olvimulogene nanivacirepvec (Olvi-Vec, referred to in previous studies as GL-ONC1, laboratory name GLV-1h68) is a modified vaccinia virus derived from the LIVP strain that contains mutations in the virus genome that allow enhanced tumor targeting (14). Pre-clinical and early-stage clinical studies found that Olvi-Vec induces both oncolysis and immune activation (14). Olvi-Vec impacts tumor growth and response to therapy through multiple mechanisms, including induction of a pro-inflammatory response, mobilization of tumor-infiltrating lymphocytes into virus-infected tumor tissues, shifting of immunosuppressive M2-type tumor-associated macrophages into immune-active M1-type, and up-regulation of the expression of genes involved in antitumor responses (13, 15). As an oncolytic immunotherapy, Olvi-Vec has been shown to be safe and mediate antitumor activity in a number of different malignancies, including platinum-resistant or -refractory ovarian cancer (PRROC) (16, 17) and advanced head and neck carcinoma (18). In preclinical studies, it has also shown antitumor effects against lung cancer (19).

The clinical activity of systemically delivered Olvi-Vec against lung malignancies was previously noted in two patients with lung metastases associated with cervical and pancreatic cancers, respectively, who received Olvi-Vec in combination with platinum-based therapy. Although these patients had been heavily pretreated and one was platinum-refractory, both experienced partial objective responses and prolonged PFS durations following Olvi-Vec monotherapy and subsequent chemotherapy was surprisingly effective (unpublished data).

We are currently conducting a phase Ib/II open-label, multicenter clinical trial in SCLC patients who have relapsed on, or were refractory to, prior platinum-based therapy. Patients received a single course of Olvi-Vec treatment and were then re-challenged with a platinum-based regimen. During this trial, we observed that Olvi-Vec treatment was associated with favorable clinical outcomes that may be due in part to restored sensitivity to platinum agents, in addition to antitumor immune activation. In this case report, we describe the disease course of a patient who showed a highly favorable response to platinum re-challenge following Olvi-Vec treatment.

## 2 Case presentation

### 2.1 Study design

The patient in this case report was enrolled in a phase Ib/II open-label, multicenter clinical trial (Olvi-Vec-SCLC-202). This trial was prospectively registered on January 12, 2023 with the Center for Drug Evaluation, Beijing, China (CTR20223399 http://www.chinadrugtrials.org.cn/clinicaltrials.searchlistdetail.dhtml) and subsequently registered on clinicaltrials.gov (NCT07136285). This trial is currently ongoing. Key inclusion criteria included adults (age ≥18 years) with histologically or cytologically confirmed SCLC, at least one measurable target lesion based on Response Evaluation Criteria in Solid Tumors (RECIST 1.1), Eastern Cooperative Oncology Group (ECOG) performance status of 0 or 1, and a life expectancy ≥6 months. Patients were required to have disease that progressed or relapsed after prior treatment with a platinum-containing chemotherapy regimen with or without other guideline-recommended therapies. Major exclusion criteria were mixed type or transformed SCLC, brain metastasis with neurological symptoms, prior or current primary malignant tumors other than SCLC, clinically significant cardiovascular disease, and oncology treatments within 4 weeks prior to the first study dose.

In the phase Ib, dose-escalating part of the trial, patients received intravenous daily doses of Olvi-Vec for 3 consecutive days. Twenty-one days after the last dose of Olvi-Vec, treatment with platinum + etoposide was initiated. The choice of platinum agent (carboplatin, cisplatin, or lobaplatin) and doses of platinum and etoposide were at the discretion of the investigator.

Antitumor response was based on a pre-specified target lesion and PFS and duration of response (DOR) as defined by RECIST 1.1. Tumor assessment visits were conducted at Week 10 and Week 16 post-initiation of the study. Subsequent assessments were conducted approximately every 9 weeks or as clinically indicated. Tumor assessments for this patient utilized enhanced computed tomography (CT) scans and were evaluated by both the investigator and a blinded independent radiology review committee. The target lesion was identified and measured by CT at baseline (prior to Olvi-Vec treatment) and at subsequent visits.

Drug safety was monitored through laboratory assessments and physical examinations. Adverse events (AEs) and their relationship to study treatment were evaluated at each clinical visit. Severity was based on the National Cancer Institute Common Terminology Criteria for Adverse Events 5.0. Potential causal relationships with Olvi-Vec were assigned by the investigator.

### 2.2 Patient description

The patient in this case report was a 58-year-old female who initially presented with a productive cough and was diagnosed with SCLC on July 12, 2023 based on imaging and tissue biopsy. Her ECOG status was 1. The target tumor size at initial diagnosis was 17.1 mm. At the time of diagnosis, the tumor stage was not evaluated. Immunohistochemical results indicated that the tumor tissue was positive for low-molecular-weight cytokeratins, thyroid transcription factor-1, CD56, chromogranin A, synaptophysin, and Ki-67 (about 60% of cells). The tissue was negative for high-molecular-weight cytokeratins 5/6 and leukocyte common antigen. Relevant biomarkers prior to first-line chemotherapy were in the normal range (**Supplementary Table 1**).

#### 2.2.1 First-line treatment

Prior to Olvi-Vec treatment, the patient received 4 cycles of first-line therapy with cisplatin + etoposide between July 29, 2023 and October 5, 2023 (**Table 1**). Tumor assessments on September 11, 2023 and October 23, 2023 found a partial response (PR) with a tumor size of 9.9 mm and 5.5 mm, respectively. She subsequently received radiotherapy to the lung (October 26 to November 18, 2023), resulting in a tumor reduction to 5.1 mm recorded on December 9, 2023, and prophylactic brain radiotherapy (December 13 to 26, 2023). Surgery was not performed, and the patient did not receive immunotherapy. Biomarkers remained normal except for human epididymis protein 4 (HE4), which was above the normal range for most of the treatment period (**Supplementary Table 1**). On October 6, 2024, a chest CT scan indicated progressive disease with a tumor size of 25.8 mm. PFS following first-line treatment was 14.3 months, DOR (defined as time from initial partial response to progression) was 13.0 months, and the initial CTFI was 12.3 months.

**Table 1:** Patient baseline characteristics, previous and trial treatments, and responses (as of 2026-03-14).

Characteristic	Case study patient
Diagnosis	
Age, years	58
Sex	Female
Target lesion size	17.1 mm
Initial treatment	
1^st^-line chemotherapy (response)	4 cycles cisplatin + etoposide (PD)
Duration of 1^st^-line platinum medication to PD (months)	
Progression-free survival	14.3 months
Duration of response	13.0 months (1^st^ PR response of 9.9 mm and best response of 5.5 mm)
Chemotherapy-free interval	12.1 months
Subsequent therapy (response)	Radical radiotherapy (best response of 5.1 mm), prophylactic brain radiotherapy
Tumor characteristics at initiation of Olvi-Vec trial	
Target lesion size at baseline prior to Olvi-Vec initiation	27.9 mm
Stage at Olvi-Vec treatment	IIIA (T3N0M0)
Treatment during Olvi-Vec trial	
Olvi-Vec total cumulative dose (single cycle of treatment)	1.5 x 10^9^ pfu (0.5 x 10^9^ pfu/day for 3 consecutive days)
Post-Olvi-Vec chemotherapy	Cisplatin 80 mg/cycle + etoposide 300 mg/cycle for 8 cycles
Response during trial	
First recorded OR based on target lesion	PR: -70.6% at day 71 post Olvi-Vec (and 2 cycles of platinum therapy in the meantime), target lesion size=8.2 mm
Best OR based on target lesion	PR: -84.6% at day 299 post Olvi-Vec, target lesion size=4.3 mm
Progression-free survival	507 days (16.7 months)
Duration of response from start of OR	436 days (14.3 months)
Current response	PD
Treatment timeline	
Diagnosis	2023-07-12
1^st^-line chemotherapy	2023-07–29 to 2023-10-05
Radiotherapy	
Lung	2023-10–26 to 2023-11-18
Brain (prophylactic)	2023-12–13 to 2023-12-26
Trial enrollment	2024-10-21
Olvi-Vec treatment days	2024-10–23 to 2024-10-25
Subsequent platinum + etoposide treatment	2024-11–16 to 2025-05-25
Last recorded scan assessment	2026-03-14

CT, computed tomography; OR, objective response; pfu, plaque-forming units; PD, progressive disease; PR, partial response.

Dates are shown as year-month-day.

#### 2.2.2 Study (second-line) treatment

**Figure 1:**
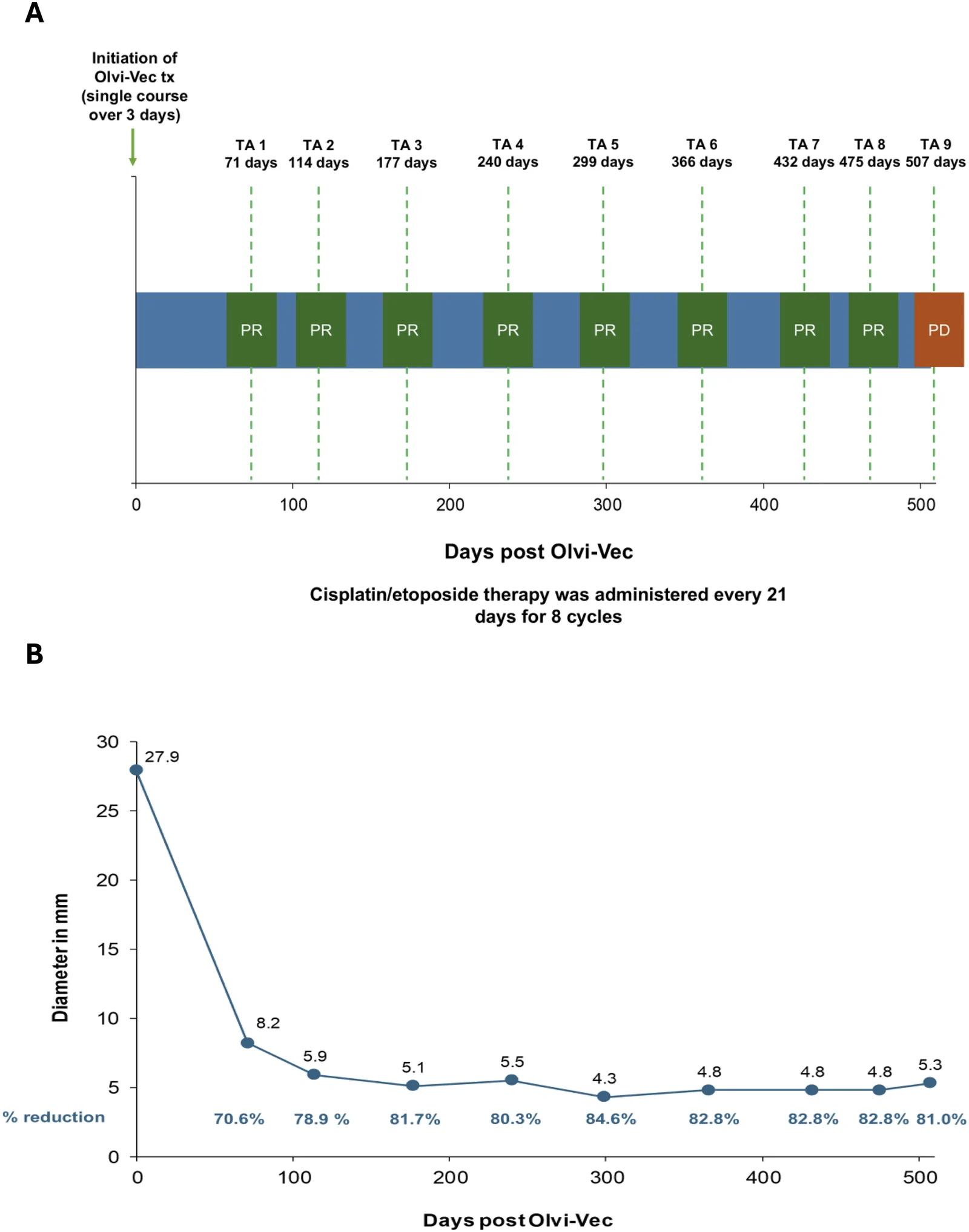
Time course of Olvi-Vec and post-Olvi-Vec platinum-based treatment and tumor response based on the target lesion measurements. **(A)** Summary of treatment course and response. **(B)** Change in diameter of target lesion during treatment. PD, progressive disease; PR, partial response; TA, tumor assessment; tx, treatment.

Following disease progression from frontline therapy, the patient enrolled in the phase Ib dose-finding portion of the Olvi-Vec-SCLC-202 trial at Zhejiang Provincial People’s Hospital (Hangzhou, Zhejiang, China) on October 21, 2024. Tumor stage, which was determined at the time of enrollment as part of the trial protocol, was stage IIIA (T3N0M0). At baseline (October 22, 2024), the patient had a 27.9 mm target lesion per RECIST 1.1 in her right lung. The patient received her first dose of Olvi-Vec on October 23, 2024. The single treatment course of Olvi-Vec was administered intravenously over 3 consecutive days at a dose of 0.5 × 10^9^ plaque-forming units (pfu) per day for a total dose of 1.5 × 10^9^ pfu (**Figure 1A**; **Table 2**). Twenty-one days after the last day of Olvi-Vec dosing, the patient was retreated with cisplatin + etoposide, the same but previously failed frontline platinum-based chemotherapy. These cisplatin + etoposide cycles continued every 3 weeks for 8 cycles (from November 16, 2024 to May 25, 2025).

**Table 2:** Adverse events of grade 3 or greater severity subsequent to Olvi-Vec therapy.

Adverse event	Event number	Severity (cell counts)	Causality to Olvi-Vec	Time from final Olvi-Vec administration	Outcome
Decreased neutrophil count	1	Grade 3 (0.88 x 10^9^/L)	Possibly	1 day	Recovered/resolved
	2	Grade 3 (0.68 x 10^9^/L)	Unlikely	6 weeks (3 weeks after 1^st^ cisplatin + etoposide cycle)	Recovered/resolved
Decreased WBC count	1	Grade 3 (1.99 x 10^9^/L)	Possibly	1 day	Recovered/resolved

WBC, white blood cell.

#### 2.2.3 Response to Olvi-Vec and post-Olvi-Vec therapy

**Figure 2:**
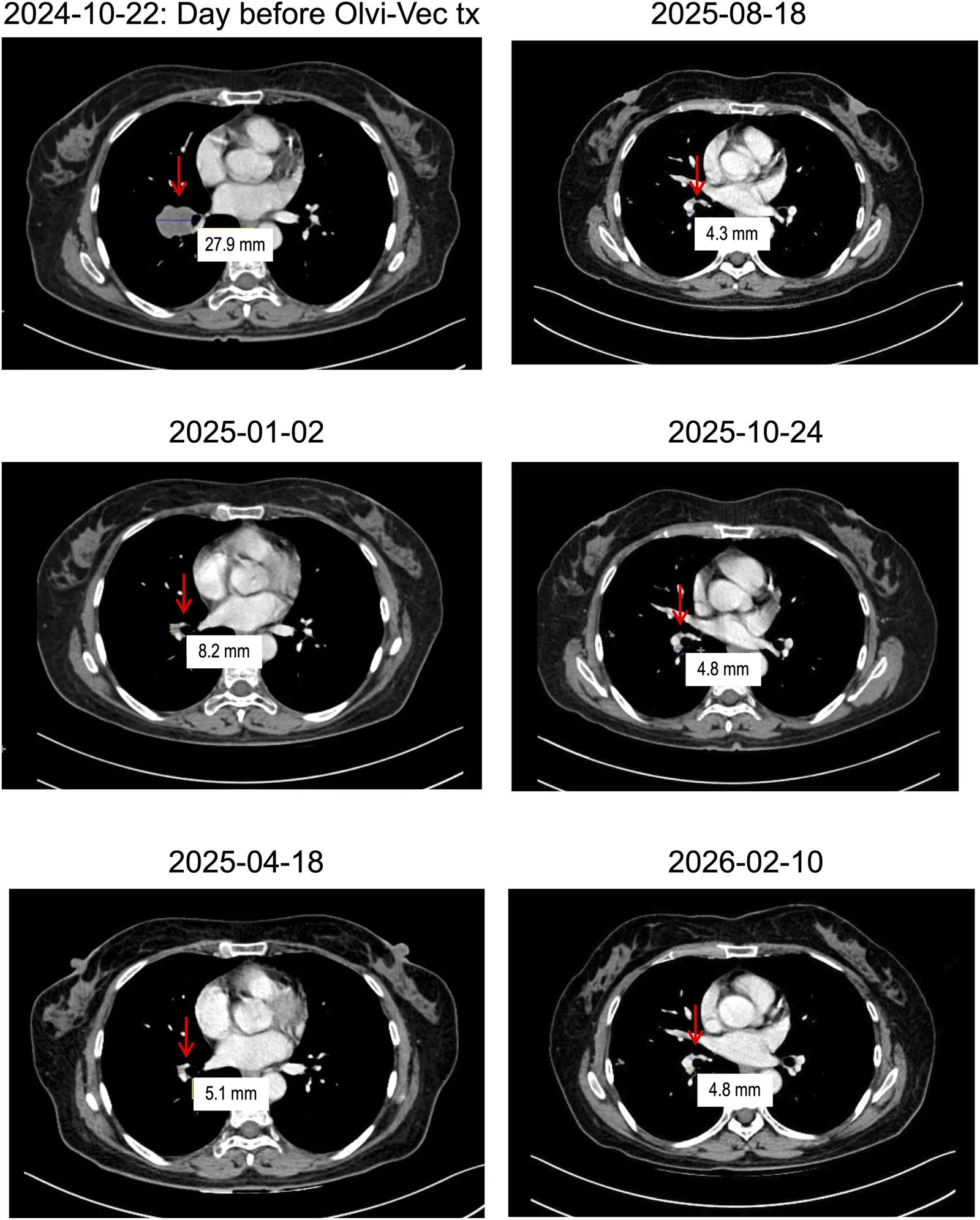
Composite of representative computed tomography scans of the target lesion before and during trial treatment. tx, treatment.

Post-Olvi-Vec tumor assessments showed an encouraging response in the target lesion compared with baseline (**Figures 1**, **2**). The patient had an initial PR at the first tumor assessment visit (January 2, 2025, day 71 after Olvi-Vec treatment and after 2 cycles of platinum therapy) with a marked decrease in tumor size from 27.9 mm to 8.2 mm (-70.6%). The diameter of the target lesion decreased further at subsequent tumor assessments. At the fifth tumor assessment on August 18, 2025 (299 days [10 months] post-Olvi-Vec initiation and after the 8th platinum cycle), the target lesion was 4.3 mm, a reduction of 84.6% from baseline (**Figures 1**, **2**). On February 10, 2026 (475 days post Olvi-Vec treatment), the tumor size was 4.8 mm (-82.8%) and it has remained at this same general size. At the most recent assessment on March 14, 2026 (507 days post Olvi-Vec), a new lesion of brain metastasis was noted, and therefore the patient was determined to have disease progression. The target lesion had increased in size slightly (to 5.3 mm) at this timepoint.

The PFS and DOR based on the most recent tumor assessment are 507 days (16.7 months) and 436 days (14.3 months), respectively. The patient received the last dose of platinum therapy on May 24, 2025 and therefore the CTFI from last dose to most recent tumor assessment is 294 days (9.7 months). Biomarkers generally showed reductions or stabilization during the first 6 months after initiation of trial treatment, although HE4 levels remained elevated (**Supplementary Table 1**).

#### 2.2.4 Safety

Olvi-Vec was generally well tolerated (**Table 2**). The only treatment-related AEs of grade 3 or greater severity were decreased neutrophils (two visits; October 26, 2024 [0.88 x 10^9^/L] and December 6, 2024 [0.68 x 10^9^/L]) and decreased white blood cell (WBC) counts (one visit; October 26, 2024 [1.99 x 10^9^/L]). Grade 3 decreased neutrophil and WBC counts were documented the day after the final Olvi-Vec administration and were judged by the investigator to be possibly related to Olvi-Vec treatment. The other occurrence of grade 3 decreased neutrophils was documented 6 weeks after Olvi-Vec administration and approximately 3 weeks after the first round of cisplatin + etoposide. This occurrence was considered possibly related to cisplatin + etoposide therapy. No modification to treatment was required, and the AEs resolved without incident. Other less severe AEs considered possibly related to Olvi-Vec administration included pyrexia and vomiting following Olvi-Vec administration (grade 1) and mild changes in blood counts and liver enzymes (**Supplementary Table 2**).

## 3 Discussion

In this case report, a patient with platinum-relapsed SCLC who received a single treatment course of Olvi-Vec followed by cisplatin + etoposide rechallenge showed a deep and durable reduction of the target tumor lesion from baseline. Although larger studies will be required to generalize this favorable observation, our data suggest that Olvi-Vec may have a role to play in the treatment of advanced SCLC, particularly in restoring responsiveness to platinum-based therapy after progression from first-line platinum-based regimens.

To put these data into perspective relative to first-line therapy, it is useful to compare the patient’s response following 4 cycles of first-line platinum (followed by radical radiotherapy) to the response following the first 4 cycles of platinum as second-line therapy subsequent to Olvi-Vec treatment. Notably, a deeper response (-78.9% vs -67.8%) was obtained after 4 cycles on platinum rechallenge following Olvi-Vec as compared to after 4 cycles of first-line platinum, an unexpected finding.

To further place the observed clinical benefit in this patient into context for second-line therapy, large studies of re-challenge with platinum-based therapy following relapse in SCLC patients have reported median PFS of approximately 5 months (7, 9, 20), even in patients considered to have platinum-sensitive relapse (9), and as low as 2.9 months in one study (21). Overall, of the four referenced studies evaluating platinum rechallenge in SCLC, the best PFS finding was from a multi-center European study, which reported a median PFS of 5.1 (95% confidence interval [CI] 4.1-5.7) months in 87 evaluable patients and 5.5 (95% CI 3.3-7.8) months in patients with a CTFI >6 months (20). In this case report, the patient’s PFS of 16.7 months following second-line therapy is longer than the 14.3 months of PFS following first-line therapy, and well exceeds the length of the highest 95% CI boundary of the historical best results (7.8 months) (20) as well as the median PFS in patients treated with first-line immune checkpoint inhibitors plus platinum (range of 4.5 to 6.4 months) (4). Additional studies will be required to verify whether the post-Olvi-Vec response observed in this single case study will occur in other patients.

Overall survival for patients with platinum-relapsed/refractory SCLC is approximately 6 months (9, 22), with patients who demonstrated a better response to first-line platinum therapy (based on longer CTFI or time to progression) showing a slight improvement in survival (approximately 1 to 2 months) following platinum rechallenge in some studies (9, 21), but not others (7, 20). The patient in this case report is alive at 19.3+ months from the start of Olvi-Vec treatment.

Although the favorable clinical response cannot be definitively attributed to Olvi-Vec followed by platinum rechallenge, the magnitude and durability of the antitumor activity observed in this patient appear greater than would be expected, and is also longer than previously observed for Olvi-Vec monotherapy. Previous early-phase studies in patients with advanced solid tumors demonstrated that intravenous Olvi-Vec monotherapy was well tolerated and associated with a virus-dose-dependent overall survival benefit, but durable objective responses were not observed (23, 24). Accordingly, this case is consistent with the hypothesis that Olvi-Vec may have contributed to the observed clinical benefit through immune activation and/or modulation of the tumor microenvironment. However, other potential explanations may include the patient’s underlying tumor biology, variability in treatment response, spontaneous disease fluctuation, or other unidentified interactions among the tumor, host, and therapy. Prospective clinical and translational studies are required to confirm the mechanism of action.

There is evidence from other studies that this case report may not simply be an outlier with a unique success story but may represent a broader population of patients who might benefit from Olvi-Vec followed by platinum therapy. In a phase II trial of patients with PRROC, intraperitoneal infusion of Olvi-Vec followed by platinum-doublet therapy with or without bevacizumab resulted in a 54% objective response rate, a 7.6-month DOR, and a median PFS of 11.0 months (16). Four patients experienced a 100% reduction in target lesions, and one platinum-refractory patient successfully received 17 cycles of platinum therapy following Olvi-Vec treatment (16). These findings exceeded historical comparisons and may potentially indicate virus-induced changes in the tumor microenvironment and clinical reversal of platinum-resistance or -refractoriness. In a recent study of PRROC patients, a large influx of T cells was observed in ascitic fluid following Olvi-Vec treatment and CD8+ tumor-infiltrating lymphocytes that penetrated into the epithelial region were identified in paired tumor biopsies. Furthermore, evaluations of tumor gene expression identified alterations consistent with immune activation and T-cell recruitment (25). Previous preclinical and clinical studies have provided a basis for potential mechanisms by which Olvi-Vec-mediated tumor re-sensitization might occur, including killing of chemoresistant cancer stem cells, enhanced infiltration of tumors with CD8+ T cells, increased expression of STAT1 and nitric oxide synthase, and augmented antigen presentation from virus-induced ICD (26). Additional studies will be required to explore these possible mechanisms. The effects of additional potential factors on response to SCLC therapies, including tumor subtype and specific genetic changes (2), are also areas of active investigation.

A limitation of this report is that it describes the clinical experience of a single patient. As such, larger clinical trials will be required to confirm and generalize these observations and gain further insights into mechanisms involved in the possible restoration of platinum sensitivity following Olvi-Vec treatment. Genetic information and data on immune cell populations and immune-related biomarkers were not available. In addition to the ongoing phase Ib/II trial in which this case report patient was enrolled, a phase I/II, open-label study of Olvi-Vec in patients with non-small cell lung cancer (NCT06463665) and a randomized phase III study in patients with PRROC (NCT05281471) are currently recruiting. These trials are expected to provide additional information on the activity and mechanisms of Olvi-Vec in patients with metastatic cancers, including effects on immune cell populations, immune-related biomarkers, and viral kinetics.

In conclusion, the case report presented here suggests that Olvi-Vec oncolytic immunotherapy may have a clinically relevant antitumor effect in some patients with SCLC, possibly due to the ability to reduce chemoresistance, which has been described as “the biggest challenge in cancer today” (11). We eagerly await results from the ongoing trial in SCLC and from larger trials in other solid tumors.

## 4 Patient perspective

The patient experienced fever and vomiting after receiving Olvi-Vec, but these symptoms subsequently resolved without any treatment. No other discomfort was associated with treatment. During Olvi-Vec treatment, the patient was pleased to remain capable of self-care and activities of daily living.

## Data Availability

The original contributions presented in the study are included in the article/**Supplementary Material**. Further inquiries can be directed to the corresponding authors.
