# Supplementary material for: Deep and durable response to re-challenge with platinum-based therapy in a platinum-relapsed small cell lung cancer patient following Olvi-Vec oncolytic immunotherapy: a case report
Source: Front Oncol. 2026 Sep 9;16:1923418. doi: 10.3389/fonc.2026.1923418 (PMC13597316; doi:10.3389/fonc.2026.1923418)
Supplement: Supplementary file 1 [file DataSheet1.pdf]

Supplementary Material

Supplementary Table 1 Biomarkers at time of diagnosis, during first-line chemotherapy, and post Olvi-Vec treatment. Red font indicates values outside the normal range. Dates are shown as year-month-day.

Bio-marker	Prior to 1st-line tx	During 1st-line tx					Post Olvi-Vec tx			Reference range
	23-07-27	23-09-09	23-12-08	24-04-26	24-06-30	24-10-06	25-04-18	25-05-23	25-07-25	
AFP, ng/mL	6.6	9.3	7.2	8.3	8.3	7.9	7.7	7.8	7.2	≤20.0
CA 72-4, U/mL	8.8	10.8	1.5	3.7	2.6	3.7	6.2	**20.0**	5.7	≤19.3
CA 125, U/mL	12.7	13.0	10.1	13.5	12.5	12.1	14.7	12.3	11.3	≤35.0
Carb ag 15-3, U/mL	5.4	9.2	5.2	5.8	5.8	5.7	5.3	6.2	4.5	≤28.5
Carb ag 19-9, U/mL	17.9	6.3	6.2	5.1	3.7	3.3	4.0	2.2	3.1	≤37.0
CEA, µg/mL	0.8	0.7	0.4	<1.7	<1.7	<1.7	<1.7	<1.7	<1.7	≤5.0
Cyto-keratin 19, ng/mL	2.5	1.4	**4.0**	1.7	2.3	2.3	2.4	3.4	2.0	≤3.8
HE4, pmol/L	57.9	**107.8**	**106.4**	96.3	84.9	86.3	**123.0**	**98.1**	**112.0**	≤92.1
NSE, ng/mL	10.8	11.3	12.8	14.3	12.9	12.3	9.4	8.3	10.9	≤20
Pro-GRP, ng/mL	16.6	8.6	8.5	11.0	18.3	**105.3**	22.9	26.6	39.1	25.0-78.0
SCC diff ag, ng/mL	1.0	0.5	2.1	1.0	0.9	0.9	1.1	0.6	1.1	≤3.0

AFP, alpha-fetoprotein; ag, antigen; CA, cancer antigen; carb, carbohydrate; CEA, carcinoembryonic antigen; diff, differentiation; GRP, gastrin-releasing peptide; HE, human epididymis; ND, not determined; NSE, neuron-specific enolase; SCC, squamous cell carcinoma; tx, treatment

Supplementary Table 2 Grade 1 and 2 adverse events post-Olvi-Vec treatment (see Table 2 in main manuscript for Grade 3 adverse events).

Time from first dose of Olvi-Vec	Adverse event	Severity grade	Causality with Olvi-Vec[a]	Outcome
Days 0 to 7	Hypoalbuminemia	1	Possibly unrelated	Recovered/resolved
	Pyrexia	1	Possibly related	Recovered/resolved
	Vomiting	1	Possibly related	Recovered/resolved
	CRP increased	1	Possibly related	Recovered/resolved
	Sinus tachycardia	1	Possibly related	Recovered/resolved
	Anemia	1	Possibly related	Not recovered/not resolved
	AST increased	1	Possibly related	Recovered/resolved
	Lymphocyte count decreased	1	Possibly related	Recovered/resolved
	Platelet count decreased	1	Possibly related	Recovered/resolved
	Lymphocyte count decreased	2	Possibly related	Recovering/resolving
Weeks 1 to 8	Constipation	1	Possibly unrelated	Not recovered/not resolved
	WBC decreased	2	Possibly unrelated	Recovered/resolved
Weeks 9 to 24	Sinus tachycardia	1	Possibly unrelated	Recovered/resolved
	Pneumonitis	1	Possibly unrelated	Not recovered/not resolved
	Asthenia	1	Possibly unrelated	Not recovered/not resolved
	Blood creatinine increased	1	Possibly unrelated	Not recovered/not resolved
	Blood uric acid increased	1 (2 events)	Possibly unrelated	Recovered/resolved (event 1) and not recovered/not resolved (event 2)
	WBC decreased	1	Possibly unrelated	Not recovered/not resolved
	Anemia	1	Possibly related	Not recovered/not resolved
	AST increased	1	Possibly unrelated	Recovered/resolved
	Neutrophil count decreased	1	Possibly unrelated	Not recovered/not resolved
	WBC decreased	2	Possibly unrelated	Recovering/resolving
	Anemia	2	Possibly related	Recovering/resolving
	Neutrophil count decreased	2	Possibly unrelated	Recovering/resolving
>24 weeks	AST increase	1	Possibly unrelated	Recovered/resolved
	Cataract	1	Definitely unrelated	Not recovered/not resolved
	Dry eye	1	Definitely unrelated	Not recovered/not resolved
	Narrow anterior chamber angle	1	Definitely unrelated	Not recovered/not resolved
	Hypoaesthesia	1	Possibly unrelated	Recovered/resolved
	Blood urea increased	1	Possibly unrelated	Not recovered/not resolved
	WBC decreased	1	Possibly unrelated	Recovered/resolved

Time from first dose of Olvi-Vec	Adverse event	Severity grade	Causality with Olvi-Vec[a]	Outcome
	Neutrophil count decreased	1	Possibly unrelated	Recovered/resolved
	Blood creatinine increased	1	Possibly unrelated	Not recovered/not resolved
	Anemia	1	Possibly unrelated	Not recovered/not resolved
	WBC decreased	2	Possibly unrelated	Recovered/resolved
	Neutrophil count decreased	2	Possibly unrelated	Recovered/resolved
	Anemia	2	Possibly unrelated	Recovering/resolving
	Blood creatinine increased	2	Possibly unrelated	Recovering/resolving

[a]As assessed by the investigator
